# Comparative Performance in the Detection of Four Coronavirus Genera from Human, Animal, and Environmental Specimens

**DOI:** 10.3390/v16040534

**Published:** 2024-03-29

**Authors:** Supaporn Wacharapluesadee, Nattakarn Thippamom, Piyapha Hirunpatrawong, Khwankamon Rattanatumhi, Spencer L. Sterling, Wiparat Khunnawutmanotham, Kirana Noradechanon, Patarapol Maneeorn, Rome Buathong, Leilani Paitoonpong, Opass Putcharoen

**Affiliations:** 1 Thai Red Cross Emerging Infectious Diseases Clinical Center, King Chulalongkorn Memorial Hospital, Bangkok 10330, Thailand; 2 Faculty of Medicine, Chulalongkorn University, Bangkok 10330, Thailand; 3 Henry M. Jackson Foundation, Bethesda, MD 20817, USA; 4 Department of National Parks, Wildlife and Plant Conservation, Ministry of Natural Resources and Environment, Bangkok 10900, Thailand; 5 Department of Disease Control, Ministry of Public Health, Muang, Nonthaburi 11000, Thailand; 6 Division of Infectious Diseases, Department of Medicine, Faculty of Medicine Chulalongkorn University, Bangkok 10330, Thailand

**Keywords:** coronaviruses, PCR, pan-CoV PCR, surveillance, reservoir host

## Abstract

Emerging coronaviruses (CoVs) are understood to cause critical human and domestic animal diseases; the spillover from wildlife reservoirs can result in mild and severe respiratory illness in humans and domestic animals and can spread more readily in these naïve hosts. A low-cost CoV molecular method that can detect a variety of CoVs from humans, animals, and environmental specimens is an initial step to ensure the early identification of known and new viruses. We examine a collection of 50 human, 46 wastewater, 28 bat, and 17 avian archived specimens using 3 published pan-CoV PCR assays called Q-, W-, and X-CoV PCR, to compare the performance of each assay against four CoV genera. X-CoV PCR can detect all four CoV genera, but Q- and W-CoV PCR failed to detect δ-CoV. In total, 21 (42.0%), 9 (18.0%), and 21 (42.0%) of 50 human specimens and 30 (65.22%), 6 (13.04%), and 27 (58.70%) of 46 wastewater specimens were detected using Q-, W-, and X-CoV PCR assays, respectively. The X-CoV PCR assay has a comparable sensitivity to Q-CoV PCR in bat CoV detection. Combining Q- and X-CoV PCR assays can increase sensitivity and avoid false negative results in the early detection of novel CoVs.

## 1. Introduction

Coronaviruses (CoVs) are enveloped, positive-sense single-stranded RNA viruses belonging to the family *Coronaviridae* (Order: *Nidovirales*). The family Coronaviridae is genetically and antigenically divided into the following four genera: Alphacoronavirus (α-CoV), Betacoronavirus (β-CoV), Gammacoronavirus (γ-CoV), and Deltacoronavirus (δ-CoV) that infect a wide variety of hosts, some of which include avian species, as well as mammals including humans, bats, swines, camels, cows, dogs, and cats [1]. α-CoVs and β-CoVs have evolved in mammalian hosts, whereas birds are the evolutionary reservoir for γ-CoVs and δ-CoVs [2].

To date, there are seven CoVs that are known to cause respiratory tract illness in humans, including the α-CoVs, human CoV (HCoV)-NL63 and HCoV-229E; and the β-CoVs, HCoV-OC43, HCoV-HKU1, severe acute respiratory syndrome coronavirus (SARS-CoV), the Middle East respiratory syndrome coronavirus (MERS-CoV), and severe acute respiratory syndrome coronavirus 2 (SARS-CoV-2) [1,3,4]. The emergence of SARS-CoV, MERS, and SARS-CoV-2 has driven a need to thoroughly understand the diversity, ecology, and evolution of coronaviruses within their natural and spillover hosts [4,5,6]. All previously documented, highly pathogenic human coronaviruses have had a zoonotic origin. SARS-CoV emerged in 2002–2003 in the Guangdong province of southern China [7]. Bats were identified as the reservoir [8,9] and SARS-CoV-like viruses were isolated from Himalayan palm civets found in a live animal market [10] and were determined to be the probable source of the outbreak. In 2012, MERS-CoV emerged in the Middle East [11]; later, camels were identified as the likely source of human infections [12]. MERS-like CoVs (bat-CoV HKU4 and HKU5) were detected in bats in China in 2004–2005 [13] and also in Thailand in 2006–2007 [14], before the primary human MERS-CoV outbreak in the Middle East and Saudi Arabia in 2012–2013 [15].

SARS-CoV-2 is a novel coronavirus that emerged in China at the end of 2019 and led to a global pandemic lasting until 2023. Until now, the origin of SARS-CoV-2 remains unclear. However, the zoonotic transmission via viral introduction through an intermediate host is the “likely to very likely” pathway, as reported by the WHO investigation team tasked with primary outbreak investigation from January to February 2020 [16]. The closest related precursor viruses are those identified in bats in Laos PDR (from *Rhinolophus malayanus*, BANAL-52) and China (*R. affinis*, RaTG13), sharing 96.8% and 96.1% whole genome sequence identity, respectively. Another example of coronavirus spillover from bats to mammals is the swine acute diarrhea syndrome (SADS-CoV) virus, which emerged in China in 2016 and 2017; the virus in swines has between 96 and 98% genome identity to bat-CoV HKU2 from horseshoe bats in China [17].

The early detection of known and unknown pathogens, including CoVs, is key to preventing and reducing transmission to animals and humans, and understanding the origin of novel viruses of pandemic potential, prior to spillover, remains essential to preventing spillover [18]. CoVs are a broad viral family with four genera and many unclassified species that may infect a wide range of animals. The molecular methods that detect a variety of CoVs from humans, domestic animals, wildlife, and environmental samples possess important distinctions that may present high rates of false negativity when used in inappropriate conditions. Pan-CoV PCR or family wide PCR assays and consensus PCR has successfully detected novel CoVs in humans, whereas the detection of unidentified or novel CoVs using only one primer set might lead to false negative reporting, due to the mismatching of primers and viral targets. Pan-CoV PCR primer design using degenerate primers to overcome this problem has largely reduced the rate of false negative reporting [19,20,21,22].

MERS-CoV was first detected from an uncharacterized viral isolate with cytopathic effects using family wide CoV PCR [11,23,24]. The first COVID-19 case outside China was detected in Thailand [25], using two pan-CoV PCR assays developed by Quan et al. [26] and Corman VM et al. [27]. Coronavirus diversity was studied in multiple host taxa from twenty countries during the USAID PREDICT project to explore the factors driving viral diversity at a global scale [28]. Two pan-CoV PCR assays targeting non-overlapping fragments of the ORF1ab were used to detect both known and novel CoVs [26,29]. A total of 19,192 samples from animals and humans (the majority coming from bats, *n* = 12,333) were assayed for the presence of CoV, 654 samples were positive using the Quan protocol and 950 using the Watanabe protocol, whereas 27% were positive for both methods from the same sample [28]. In total, 100 discrete phylogenetic clusters were identified from sequences represented in this study, 91 of which were found in bats. Ecological and epidemiologic analyses showed that CoV diversity patterns correlate with bat diversity and also that bats are the major evolutionary reservoirs and ecological drivers of CoV diversity. However, avian species were not included in this PREDICT study and more investigation is required to assess their impact on CoV diversity.

Most of the pan-CoV PCR protocols target the amplification of CoVs belonging to the genera α-CoV and β-CoV, while fewer protocols amplify the genera γ-CoV and δ-CoV [20]. The circulation of γ-CoV and δ-CoV in mammals might be underestimated, due to the use of primers that cannot amplify γ-CoV and δ-CoV. To overcome this problem, a pan-CoV protocol that can detect all four CoV genera with high sensitivity has been developed to account for the presently described viral diversity, including recently discovered novel CoVs [19,20,21,22].

In this study, we selected the two standardized PREDICT pan-CoV protocols [28], including Quan’s protocol (called Q-CoV PCR) [26] and Watanabe’s protocol (W-CoV PCR) [29], and the pan-CoV assay developed by Xiu L. et al. (X-CoV PCR) [21] to evaluate the performance and limitations on the detection of four CoV genera from different sources of hosts and specimens.

## 2. Materials and Methods

### 2.1. Specimens Used in Assay Comparison

Human specimens: Archived upper respiratory specimens submitted to the Emerging Infectious Diseases Clinical Center (EIDCC) laboratory, King Chulalongkorn Memorial Hospital from October 2022 to December 2023 were included in this study. Specimens were previously tested using a validated real-time PCR assay for SARS-CoV-2 (GenePath Dx CoViQuick v2.0, Ankara, Turkey) and 33 other respiratory pathogens (FTD^®^ Respiratory Pathogens 33 Assay, LABGENE SCIENTIFIC SA ZI, Châtel-Saint-Denis, Switzerland). The panel of samples was tested using three established pan-CoV PCR protocols, including Q-CoV PCR [26], W-CoV PCR [29], and X-CoV PCR [21].

Wastewater specimens: Archived wastewater specimens submitted to the EIDCC laboratory from July 2022 to December 2023 were included in the study. Specimens were previously tested for SARS-CoV-2 using a validated real-time PCR assay (GenePath Dx CoViQuick v2.0). Positive and negative SARS-CoV-2 samples were tested using the three pan-CoV PCR assays.

Animal specimens: Archived positive bat α-CoV and β-CoV specimens tested using Q-CoV PCR were included to compare the performance with W-CoV and X-CoV PCRs. Additionally, archived positive γ-CoV and δ-CoV specimens that tested positive using X-CoV PCR were included, to compare the performance with Q-CoV and W-CoV PCRs.

### 2.2. RNA Extractions and cDNA Synthesis

Viral RNA from nasopharyngeal swabs from humans, as well as rectal and oral swabs from bats and birds, was extracted with the MagPurix^®^ Viral RNA Extraction Kit (Zinexts Life Science Corp., New Taipei, Taiwan). Viral RNA from wastewater samples was extracted with the ZR Urine RNA Isolation Kit™ (Zymo Research, Murphy Ave, Irvine, CA, USA), as previously published [30]. Briefly, 40 mL of wastewater was transferred to a fresh 50 mL centrifuge tube containing glass beads (HiMedia, Kennett Square, PA, USA) and was homogenized using a sample disruption instrument (FastPrep-24TM 5G, MP Biomedicals, Irvine, CA, USA) for 60 s at 6 m/s. The supernatant was transferred to a new tube and filtered through a 1.6 µm pore size glass fiber filtration membrane (ZRC GF™ Filter, Zymo Research, Murphy Ave, Irvine, CA, USA) for RNA enrichment. In total, 700 µL Urine RNA Buffer was applied to the filter and the flow-through was collected in a nuclease-free tube. RNA was purified with a Zymo-Spin™ IC Column (Zymo Research, Murphy Ave, USA), following the manufacturer’s protocol, and was eluted with 50 µL of DNase/RNase-Free Water. The RNA was immediately prepared for SARS-CoV-2 real-time PCR assay and was kept at −80 °C for further analysis.

cDNA was synthesized using the Superscript III First-Strand Synthesis System (Thermo Fisher Scientific, Waltham, MA, USA) following the manufacturer’s protocol. Briefly, 1 µL of 50 ng/µL random hexamers and 1 µL dNTPs (10 mM each) were mixed with 8 µL of extracted RNA and incubated at 65 °C for 5 min. The resulting reaction was then combined with 10 µL of a mixture of 4 µL 5X Firststrand buffer, 4 µL of 25 mM MgCl_2_, 2 µL of 100 mM DTT, 1.0 µL of Ribonuclease Inhibitor (40 U/µL), and 1.0 µL of Superscript III Reverse Transcriptase (200 U/µL). Reverse transcription was performed via incubation for 10 min at 25 °C, 50 min at 50 °C, 5 min at 85 °C, and held at 4 °C indefinitely. The cDNA was stored at −80 °C until used for PCR assays.

### 2.3. Pan-Coronavirus PCR Protocols Selected for Comparison

**Figure 1:**
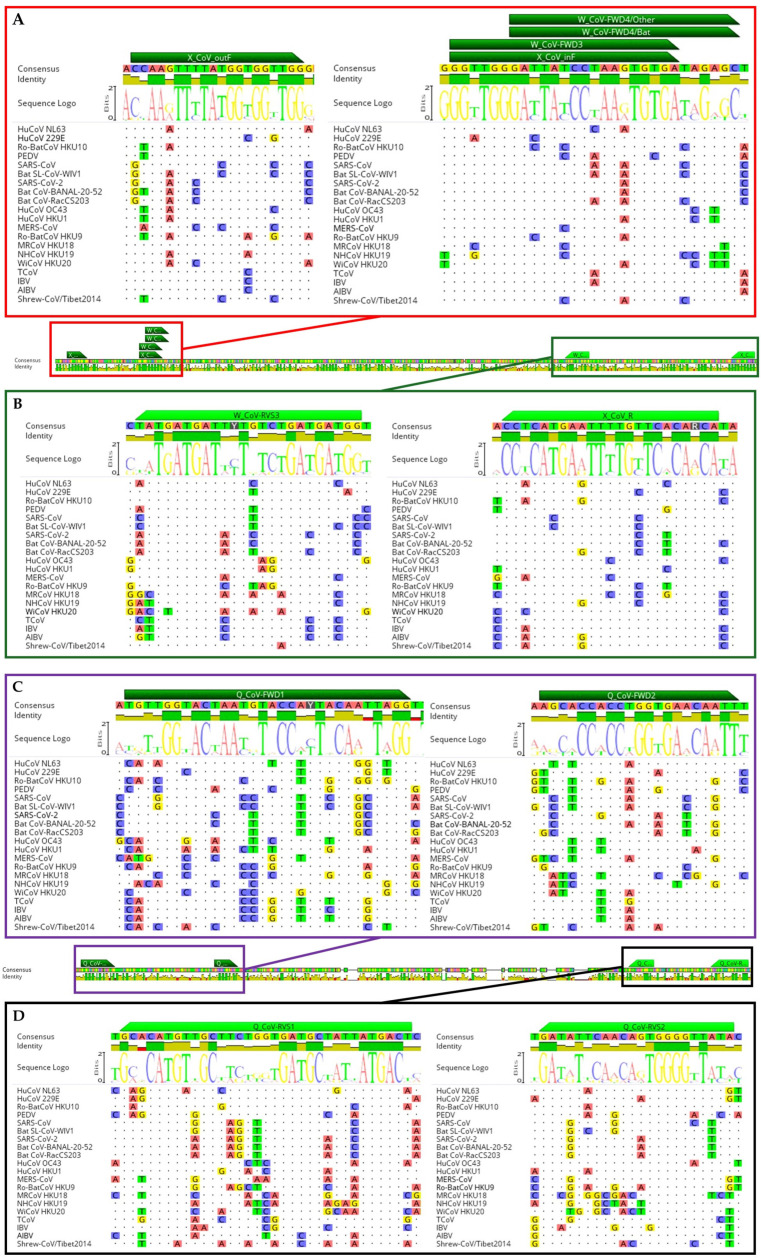
The primer alignment with 70 CoV sequences represents all four genera of CoVs. (**A**,**B**) The target regions of W-CoV PCR and X-CoV PCR were at position 14,370–14,750 and 14,255–14,927, respectively (aligned to HCoV-229E, accession No. NC_002645.1). (**C**,**D**) The target regions of Q-CoV PCR were at position 17,480–17,820. The figures were created using Geneious Prime^®^ 2024.0.2 (Auckland, New Zealand).

Three established PCR protocols were used in this study to compare their sensitivity, specificity, and limitations, including two protocols that had been used in the PREDICT project from 2009 to 2020, which were modified from Quan [26] and Watanabe [29], as well as newly designed primers (from 2020) by Xiu et al., 2020 [21]. All three protocols targeted amplification in the RNA-dependent polymerases (RdRp) region; the details of primers and the PCR profile are shown in Supplementary Table S1 and the primer map is shown in Figure 1. The protocol consisted of nested or hemi-nested RT-PCR. The first step PCR reaction mixture contained 2 µL of cDNA, 2.5 µL of 10X PCR buffer, 0.75 µL of 50 mM magnesium choline, 0.5 µL of 10 mM dNTP mix, 1 µL of each primer (20 µM), 0.1 µL of Platinum™ Taq DNA Polymerase, and nuclease-free water, with a final volume of 25 µL for each reaction. The second amplification was performed using nested primers (for the Q-CoV PCR protocol) and hemi-nested primers for the W-CoV PCR and X-CoV PCR protocols, as follows: 1 µL of PCR product, 2.5 µL of 10X PCR buffer, 0.75 µL of 50 mM magnesium choline, 0.5 µL of 10 mM dNTP mix, 1 µL of each primer (20 µM), 0.1 µL of Platinum™ Taq DNA Polymerase, and nuclease-free water, with a final volume of 25 µL for each reaction.

The thermal cycling profile for the first and second rounds of PCR can be found in Supplementary Table S1. Positive and negative controls were contained in each run of the assay to ensure test validity. The PCR products were electrophoresed and visualized using capillary electrophoresis (QIAxcel system, Qiagen, Hiden, Germany) or a 2% agarose gel, together with a 100 bp DNA ladder at 120 V for 30 min. The positive nested amplicons were Sanger sequenced for CoV species confirmation by sending the purified PCR product to the 1st BASE Company (Malaysia).

### 2.4. Statistical Analysis

Assay sensitivity for each viral genus and each sample type was calculated relative to the “gold standard” tests, including q-RT-PCR for human and wastewater samples, the “Q” protocol for bat specimens, and the “X” protocol for avian specimens. Comparisons of the sensitivity between each tested method within the specific sample types were investigated using a Chi-squared test for two proportions [31]. The rates of positivity for each testing method using CoV genus were compared using a Test for Equality of Several Population Proportions [32]. Results were considered statistically significant at the 95% confidence level.

## 3. Results

### 3.1. Clinical and Environmental Specimens Used in Evaluating the Assay

This study included ninety-five (95) positive CoV samples confirmed using real-time PCR or sequence comparison from pan-CoV PCR, and forty-six (46) negative specimens. Table 1 summarizes the coronavirus-positive specimens used to evaluate three pan-CoV PCR assays.

Human specimens: A total of fifty (50) samples were assessed, including seven (7) positive specimens for SARS-CoV-2, seven (7) of HCoV-OC43, five (5) of HCoV-229E, three (3) of HCoV-NL63, one (1) of HCoV-HKU1, and twenty-seven (27) negative samples were confirmed using both the SARS-CoV-2 and 33 respiratory pathogen assays.

Wastewater specimens: A total of forty-six (46) samples were previously tested for SARS-CoV-2 using real-time PCR and were used for evaluating the pan-CoV PCR assays. Twenty-seven (27) samples were SARS-CoV-2 positive, and nineteen (19) were SARS-CoV-2 negative.

Animal specimens: Twenty-eight (28) CoV positive from bat rectal swabs using the Q-CoV PCR assay for α- or β-CoV were used to evaluate the W- and X-CoV PCR assays. Seventeen (17) CoV positive samples from birds for γ- or δ-CoV using X-CoV PCR were used to assess the performance of Q- and W-CoV PCR assays.

Supplementary Tables S2–S5 summarize the PCR results and the characteristics of the samples, including the collection date, source of specimens, and list of animal species.

### 3.2. Comparative Analysis of Different Pan-CoV PCR Assays in Human Specimens

Among fifty (50) human clinical samples tested using three separate pan-CoV protocols, 21 (42.0%), 9 (18.0%), and 21 (42.0%) specimens were detected using Q-, W-, and X-CoV PCR assays, respectively (Table 2 and Table S2). Q-CoV PCR showed concordant results with real-time PCR on the detection of SARS-CoV-2, HCoV-OC43, HCoV-229E, and HCoV-HKU1; two of the three false negative results arose from non-detection of HCoV NL63 (Supplementary Table S2). The X-CoV PCR assay showed concordant results with real-time PCR on the detection of SARS-CoV-2, HCoV-229E, HCoV-HKU1, and HCoV-NL63, with two of the seven HCoV OC43 samples showing discordant results. Q-CoV PCR and X-CoV-PCR demonstrated concordant sensitivity for the detection of SARS-CoV-2 (Ct range 24.7–30.86), HCoV-229E (Ct range 12.8–28.07), and HKU1 (Ct 23.4). Two HCoV-OC43-positive samples with Ct 28.65 and 31.16 tested positive using Q-CoV-PCR, but negative using W- and X-CoV PCR assays. On the contrary, two HCoV-NL63 samples with Ct 29.51 and 29.5 tested negative using Q-CoV PCR, but positive using X-CoV PCR (Table S2). However, HCoV-NL63 with Ct 20.99 showed positive results using both Q- and X-CoV PCR assays. The W-CoV PCR assay was unable to detect SARS-CoV-2, HCoV-HKU1, and HCoV-NL63, but was able to detect five out of seven HCoV-OC43 and four out of five HCoV-229E; those real-time PCR Ct values were less than 28.13 and 27.15, respectively (Table S2). All twenty-seven human CoV-negative samples resulted in concordant results using all three pan-CoV assays used in this study.

### 3.3. Comparative Analysis of Different Pan-CoV PCR Assays in Wastewater Specimens

Forty-six (46) wastewater specimens included for assay evaluation were previously tested for SARS-CoV-2 using real-time PCR. The specimens were collected from either hospital (*n* = 23) wastewater from July 2022 to May 2023 or aircraft (*n* = 23) wastewater from October to November 2023. Twenty-seven samples were determined to be SARS-CoV-2 positive using real-time PCR, including 21 from hospital and 6 from aircraft wastewater specimens. Overall, 30 of 46 (65.22%), 6 of 46 (13.04%), and 27 of 46 (58.70%) specimens were deemed to be CoV positive using Q-, W-, and X-CoV PCR assays, respectively (Table 2). Among the pan-CoV assays, nine SARS-CoV-2, seven HCoV-229E, and fourteen HCoV-OC43 were detected using Q-CoV PCR (Table S3); 11 SARS-CoV-2, 3 HCoV 229E, and 13 HCoV OC43 were detected using X-CoV PCR; and 2 HCoV 229E and 4 HCoV OC43 were detected using W-CoV PCR (Table S3). We observed that the samples with real-time Ct values less than 31 showed concordant results between SARS-CoV-2 real-time PCR and the pan-CoV PCR assays. However, other CoV species, such as HCoV OC43 and HCoV 229E, were detected using pan-CoV PCR assays instead of SARS-CoV-2, when a low amount of SARS-CoV-2 (Ct values > 31) was present in the same sample (Table S3). Multiple CoVs in one sample were detected in seven specimens when results from two to three pan-COV PCR assays were combined. W-CoV PCR was unable to detect SARS-CoV-2, but could detect additional non-SARS-CoV-2 that was co-circulating in wastewater from the SARS-CoV-2-positive samples using Q-CoV-PCR, including four HCoV-229E and two HCoV-OC43.

### 3.4. Comparative Analysis of Different Pan-CoV PCR Assays in Bat Specimens

Twenty-eight Q-CoV PCR-positive samples (α-CoV and β-CoV) from bat rectal swab specimens were selected for comparison of the sensitivity and specificity with W- and X-CoV PCR assays, including 16 α-CoVs and 12 sarbecovirus (Table S4). Sixteen samples (57.1%) tested positive for α-CoVs using W-CoV PCR, with 14 demonstrating positive concordant results with Q-CoV-PCR, while the other two tested negative. The remaining two α-CoV positive samples, when tested using W-CoV, were identified as β-CoV using the Q-CoV PCR assay. However, the W-CoV PCR assay failed to detect any bat sarbecovirus (β-CoV) in this study, while it detected α-CoV co-infection within β-CoV-positive samples.

In total, 26 of 28 CoV positive samples using Q-CoV PCR were positively detected using the X-CoV PCR assay, including 10 of 12 sarbecovirus and all 16 α-CoVs. Twenty-four samples demonstrated concordant results between the Q- and X-CoV PCR assays. Two negative samples were detected using X-CoV PCR, including a sarbecovirus and Alphacoronavirus HlYN10; however, X-CoV PCR detected these two strains of CoV in the other specimens. In addition, two positive samples using X-CoV PCR identified different CoV strains from Q-CoV PCR, as follows: Alphacoronavirus YN2012 vs. Alphacoronavirus HlYN10 and Alphacoronavirus HlYN10 vs. Sarbecovirus Ra22QT77, respectively (Table S4). Similar to the results from wastewater, co-positivity was detected in four specimens, when the results from each pan-CoV PCR assay were combined.

### 3.5. Comparative Analysis of Different Pan-CoV PCR Assays in Avian Specimens

In total, 17 X-CoV PCR-positive samples from avian oral and rectal swab specimens (7 of γ-CoV and 10 of δ-CoV) were selected for comparison of the sensitivity with Q-CoV PCR and W-CoV PCR methods (Table S5). Q-and W-CoV PCR failed to detect δ-CoVs from all selected avian samples in this study. Q-CoV PCR was able to detect γ-CoV from one (1/17, 5.88%) sample and the sequencing result characterized it as a pigeon-dominant coronavirus (Table S5). Four of seven samples tested positive for γ-CoV with the W-CoV PCR assay; the sequence results were compatible with the X-CoV PCR assay, including three Anser fabalis coronavirus NCN2 and one Avian coronavirus (Table S5).

### 3.6. Comparative Analysis of Different Pan-CoV PCR Assays in CoV Detection from Each Genus

**Figure 2:**
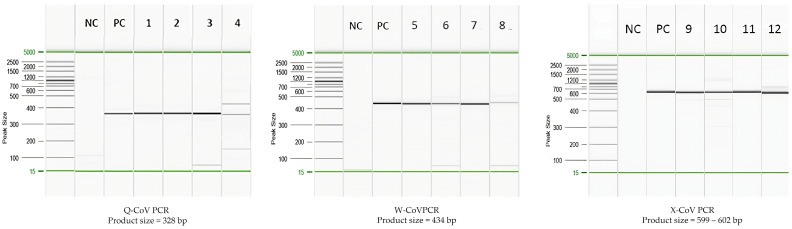
Capillary electrophoresis results of PCR products using the QIAxcel system. From three pan-CoV PCR protocols using QIAxcel. M: the QIAxcel size marker, 15–5000 bp; Lanes 1–4: the assay was performed using Q-CoV PCR and positive samples were alphacoronavirus from bat rectal swab, Pigeon-dominant Coronavirus from pigeon oral swab, HCoV-229E from human nasopharyngeal swab, and SARS-CoV-2 from wastewater sample, respectively. Lanes 5–8: the assay was performed using W-CoV PCR and positive samples were alphacoronavirus from bat, Anser fabalis coronavirus NCN2 from duck rectal swab, HCoV-229E from human nasopharyngeal swab, and HCoV-OC43 from wastewater sample, respectively. Lanes 9–12: the assay was performed using X-CoV PCR and positive samples were alphacoronavirus from bat, gammacoronavirus from Little Egret, HCoV-229E from human nasopharyngeal swab, and HCoV-229E from wastewater sample, respectively. NC = Negative control. PC = Positive control.

**Figure 3:**
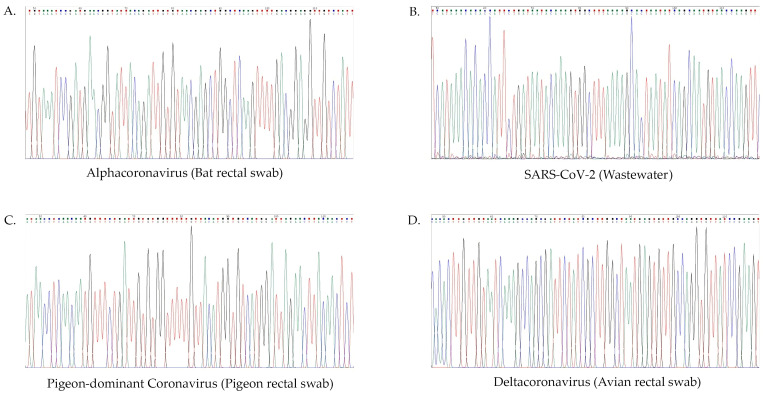
The chromatogram of the positive PCR product amplified using (**A**) W-CoV PCR, (**B**) X-CoV PCR, (**C**) Q-CoV PCR, and (**D**) X-CoV PCR for bat alphacoronavirus, SARS-CoV-2, pigeon-dominant coronavirus, and deltacoronavirus, respectively. The specimen source is in parentheses.

The size of PCR products from Q-, W-, and X-PCR was 328, 434, and 599–602 bp, respectively (Figure 2). The chromatogram from the Sanger sequencing of α-, β-, δ-, and γ-CoV showed clear peaks (Figure 3). The rates of positivity in three CoV-PCR protocols in each CoV genus were analyzed and are summarized in Table 2. The Q and X protocols were not significantly different in their rates of positivity for the detection of CoV in human, wastewater, and bat samples (*p* = 1.000, 0.5218, and 0.1536, respectively), but were significantly different for avian specimens (100% positive using the X protocol, *p* < 0.0001), for which Q and W had 5.88 and 23.53% rates of positivity, respectively. The Test for Equality of Several Population Proportions (Q:W:X assay) results that compare the rates of positivity are included in Table S7. Our study showed that Q, W, and X protocols performed comparably (*p* = 0.556) for the detection of α-CoVs, with overall rates of positivity for each protocol at 20.57%, 15.60%, and 18.44%, respectively. Among the β-CoVs, the W protocol (6.38% positive rate) had significantly lower rates of detection than the Q and X protocols (35.46% and 34.04%, respectively) (*p* < 0.0001) (Table 3). The positive rates of the three assays were not statistically significant when detecting γ-CoVs (*p* = 0.099), whereas there were significantly different rates of detection for δ-CoVs (*p* < 0.0001). However, only 7 and 10 samples of γ-CoV and δ-CoV, respectively, were available to evaluate, and further investigations using a larger sample size are needed. Overall, the X protocol showed a more consistent sensitivity across all source species tested than the other two protocols.

## 4. Discussion

In this study, the detection performance for four genera of coronaviruses using three established pan-CoV PCR assays was compared using clinical and biological samples from humans, animals, and the environment. All assays target the RdRp gene on the difference region with some primer overlap (Watanabe’s and Xiu’s protocols in their nucleotide sequences) (Table S1). The coronavirus RdRp is highly conserved among the different CoVs and provides sequences of sufficient length for phylogenetic analysis and a preliminary classification [33]. CoV PCR-positive samples for all four CoV genera from humans, bats, avians, and wastewater samples were included in the study to assess each protocols performance for each genus (Table 1).

Most of the pan-CoV PCR evaluation studies were performed using a single PCR method [19,21,22]. Alternatively, head-to-head comparisons of the sensitivity and rates of positivity for three assays were conducted in this study, which aimed to select appropriate assays for CoV detection in different hosts/sources of specimens and within the CoV genera. When three pan-CoV PCR assays were compared among five HCoV species in clinical specimens, the positive rate for each virus was different depending on the assay used. Among the human CoVs from twenty-three (23) positive human clinical samples using three separate pan-CoV protocols, 21 (91.3%), 9 (39.1%), and 21 (91.3%) specimens were detected using Q-, W-, and X-CoV PCR assays, respectively (Table 2). Q-CoV PCR showed a higher positive rate than W- and X-CoV PCR for HCoV-OC43 detection (Table S2). The primer mismatches of three assays for HCoV-OC43 were similar (0–2), but the PCR product amplified using Q-CoV PCR (328 base pair) was shorter than others (434 and 602 for W- and X- CoV PCR) (Table S1), which caused a superior sensitivity than the assay with larger PCR products. The positive rate of HCoV-NL63 detection using X-CoV PCR (3 of 3) is higher than Q- (1 of 3) and W-CoV PCR (0 of 3). The primer mismatch of X-, Q-, and W-CoV PCR were 0–1, 2–5, and 0–7, respectively, which is assumed to have a significantly large impact on the priming efficiency and PCR sensitivity [34]. However, a limited number of clinical samples was used for evaluation in our study.

The Q-CoV PCR primers were designed in 2010 to detect bat SARS-like CoV using degenerate primers [26]. Despite 2–4 primer mismatches to SARS-CoV-2 (Table S6), this protocol successfully detected the first COVID-19 case in Thailand [25], while the X-CoV PCR primers were newly designed in 2020 and have a 0–1 primer mismatch to SARS-CoV-2. Both assays have equal sensitivity at PCR Ct values less than 31 in human specimens (Table S2). At a low copy number of SARS-CoV-2 (Ct > 30) in wastewater specimens, the X-CoV PCR showed a higher sensitivity than the Q-CoV PCR assay (Table S3), which is hypothesized to be due to the number of primer mismatches. The W-CoV PCR could not detect SARS-CoV-2 in clinical samples and laboratory strains due to eight primer mismatches within the reverse primers [19,25], as confirmed in our study.

Interestingly, the pan-CoV PCR results in wastewater showed discrepancies from the SARS-CoV-2 real-time PCR when the Ct values were higher than 31. HCoV-OC43 and HCoV-229E were detected using Q-, W-, and X-CoV PCR assays (Table S3) from the SARS-CoV-2 real-time PCR positive samples. The wastewater, where the pooled samples contained multiple pathogens from many sources, and pan-CoV PCR was able to amplify all four CoV genera, of which the primers preferentially bind to viral RNA at higher concentrations and with a higher compatibility (i.e., less primer mismatches) between primers and viral targets. However, sequence analysis and interpretation of the mixed circulation of coronavirus within the same samples should be performed carefully and should consider the inputs and primers used. Overlapping sequencing peaks should be further analyzed by cloning prior to sequencing, to identify possible hidden or low-concentration viral sequences [35].

We used twenty-eight bat coronavirus-positive samples, tested using Q-CoV PCR, our routine “gold-standard” protocol for bat coronavirus detection, to compare the assay sensitivity with the W-CoV and X-CoV PCR assays. Two samples, a sarbecovirus and alphacoronavirus HIYN10, were not detected using X-CoV PCR, which Q-CoV PCR had previously detected. Primer mismatches are not believed to be the cause of these discordant results, as these two virus strains were detected from other specimens (Table S4). Q-CoV PCR showed a higher sensitivity than X-CoV PCR, because the Q-CoV PCR uses a nested PCR platform, whereas X-CoV PCR uses a hemi-nested PCR and amplifies a shorter PCR amplicon (328 and 602 base pairs, respectively). All concordant bat α-CoVs (14/16) detected using the W-CoV PCR assay were identical to the assigned Q-CoV-PCR sequences. Q-CoV PCR showed a higher sensitivity than W-CoV PCR for the detection of the HIYN10 virus. Unfortunately, HIYN10-positive samples have a limited volume with limited viral RNA and do not have bat viral isolates to conduct a quantitative sensitivity test.

W-CoV PCR was unable to detect bat sarbecoviruses in this study (*n* = 12). It should be noted that W-CoV PCR was previously able to amplify different strains of β-CoVs, such as bat CoV HKU9 from *Pteropus lylei* [36], as well as multiple strains from multiple bat species from PREDICT [28]. Recently, the modified Watanabe primers were re-designed and showed the ability to detect four genera of CoVs [19]. However, two sarbecovirus-positive samples using Q- and X-CoV PCR were positive for Bat alphacoronavirus RaYN17 using W-CoV PCR assay. Multiple infections by different virus strains or genus were found in four bat specimens depending on the PCR method used; using one PCR method may lead to a misdiagnosis or incomplete diagnosis of multiple infections. Moreover, using the Q- and X- PCR assays, we were able to identify multiple CoV infections with α- and β-CoV (sample no. B20) and two α-CoV strains (sample no. 14). The study of bat-CoV diversity should consider using a strategy that detects and differentiates a variety of CoV strains and more than one PCR assay that uses different primer positions to avoid misinterpretation. In the CoV diversity study throughout the PREDICT project, 27% of samples were positive using both the Q- and W-protocols, and Q and W could identify different viral strains and genera [28].

To test the ability of Q- and W- CoV PCRs to detect γ- and δ-CoVs, avian-positive specimens (*n* = 17) using X-CoV PCR were used. It was not a surprise that the Q-and W-CoV PCR assays could not detect δ-CoV because of substantial primer mismatches (six and five nucleotides, respectively) found in the reverse primer used in the nested PCR step (Table S6), which prevented sufficient primer annealing. A higher number of primer mismatches affected the detection limit of the virus in Watanabe primers [19]. In contrast, a 0–1 mismatch was found in all three primers of X-CoV PCR for the δ and γ-CoV genus. W-CoV PCR was able to identify two of four γ-CoV strains, tested positive using X-CoV PCR. Similarly, Q-CoV PCR was able to detect Pigeon-dominant Coronavirus, but failed to detect the other three γ-CoV strains that tested positive using X-CoV PCR. These results confirm that Q- and W-CoV PCR were not suitable to use for the detection of δ-CoV and γ-CoV. More specimens are needed to confirm this conclusion, due to the limited number of samples used in our study.

From our study, the total sensitivity % showed that either Q-CoV or X-CoV PCRs are viable options for studying coronavirus diversity in humans and bats. However, from individual analysis, X-CoV PCR was the method of choice for γ-CoV and δ-CoV testing. X-CoV PCR should be used for the study of CoVs from the environment, including wastewater or air samples that may contain CoV from birds or swines [21,37]. To avoid false negative results, the use of two protocols during testing is recommended. From our study, a combination of Q-and X-PCR methods can increase the sensitivity and reduce the reporting of false negative results.

## 5. Conclusions

We evaluated three previous pan-CoV PCR assays on the same samples to compare their sensitivity and performance in detecting four CoV genera. X-CoV PCR can detect all four CoV genera. Two methods (Q-CoV PCR and W-CoV PCR) were developed in 2010 and were used during the multi-national PREDICT project. In contrast, the primers for the X-CoV PCR protocol were designed in 2020 to account for two emerging human CoVs, MERS and SARS-CoV-2, during primer design. X-CoV PCR showed a higher sensitivity and specificity for SARS-CoV-2 detection than the W and Q assays. The evaluation of results from a variety of sample sources and CoV genera from our study can be used as a guide to select the optimal method for future studies. However, using two protocols that amplify non-overlapping fragments of the RdRp gene should be considered for detecting novel CoV and studying CoV diversity to avoid false negatives from primer mismatches with novel viruses.

## Figures and Tables

**Table 1:** List of coronavirus-positive specimens used for evaluation of three pan-CoV PCR assays.

Virus Name	CoV Genus	Host/Source	Sample Type	Confirmed Assay	Number
HCoV-229E	α CoV	Human	NPS *	Real-time PCR	5
HCoV-NL63	α CoV	Human	NPS	Real-time PCR	3
Bat Alphacoronavirus HKU10	α CoV	Bat	Rectal swab	Q-CoV PCR and sequencing	14
Bat Alphacoronavirus RsYN14	α CoV	Bat	Rectal swab	Q-CoV PCR and sequencing	2
HCoV- HKU1	β CoV	Human	NPS	Real-time PCR	1
HCoV-OC43	β CoV	Human	NPS	Real-time PCR	7
SARS-CoV-2	β CoV	Human	NPS	Real-time PCR	7
SARS-CoV-2	β CoV	Hospital, aircraft	Wastewater	Real-time PCR	27
Bat Sarbecovirus isolate Ra22QT77	β CoV	Bat	Rectal swab	Q-CoV PCR and sequencing	12
Anser fabalis coronavirus NCN2	γ CoV	Avian	Oral swab Rectal swab	X-CoV PCR and sequencing	3
Avian coronavirus Glaucous-winged gull CIR-66002	γ CoV	Avian	Oral swab Rectal swab	X-CoV PCR and sequencing	1
Avian coronavirus isolate CoV-9518-2016	γ CoV	Avian	Oral swab Rectal swab	X-CoV PCR and sequencing	2
Pigeon-dominant Coronavirus	γ CoV	Avian	Oral swab	X-CoV PCR and sequencing	1
Night-heron coronavirus HKU19	δ CoV	Avian	Rectal swab	X-CoV PCR and sequencing	1
Magpie-robin coronavirus HKU18	δ CoV	Avian	Oral swab Rectal swab	X-CoV PCR and sequencing	2
unclassified Deltacoronavirus	δ CoV	Avian	Rectal swab	X-CoV PCR and sequencing	7

* Nasopharyngeal swab.

**Table 2:** Comparative sensitivity among Q-CoV (Q), W-CoV (W), and X-CoV (X) PCR assays in each CoV genus from different sources of sample.

	Number of PCR Positive Samples Using Three PCR Assays in Four Sample Sources											
CoV Genus	Human			Water			Bat			Avian		
	Q	W	X	Q	W	X	Q	W	X	Q	W	X
α	6	4	8	7	2	2	16	16	16	0	0	0
β	15	5	13	23	4	25	12	0	10	0	0	0
γ	0	0	0	0	0	0	0	0	0	1	4	7
δ	0	0	0	0	0	0	0	0	0	0	0	10
Negative no.	29	41	29	16	40	19	0	12	2	16	13	0
Positive no.	21	9	21	30	6	27	28	16	26	1	4	17
Total	50	50	50	46	46	46	28	28	28	17	17	17
% positive	42.00	18.00	42.00	65.22	13.04	58.70	100.00	57.14	92.86	5.88	23.53	100.00
95% CI	79.79–100%	19.18–59.08%	79.79–100%	GS	5.69–34.31%	79.26–100%	GS	38.83–75.47%	83.32–100%	0.00–17.07%	3.34–43.69%	GS

GS = Gold standard assay.

**Table 3:** Comparative sensitivity among Q-CoV, W-CoV, and X-CoV PCRs in each CoV genus.

CoV Genus	Number (%) Positive/Negative Detected Using Three Assays		
	Q-CoV PCR	W-CoV PCR	X-CoV PCR
α	29 (20.57)	22 (15.60)	26 (18.44)
β	50 (35.46)	9 (6.38)	48 (34.04)
γ	1 (0.71)	4 (2.84)	7 (4.96)
δ	0 (0.00)	0 (0.00)	10 (7.09)
Negative	61 (43.26)	106 (2.84)	50 (35.46)
Total	141	141	141
Positive	80	35	91
% positive	56.74	24.82	64.54

## Data Availability

Data are contained within the article or Supplementary Material.

## Supplementary Materials

The following supporting information can be downloaded at: https://www.mdpi.com/article/10.3390/v16040534/s1. Table S1: Pan-CoV primers and PCR protocol used in this study; Table S2. Comparative results of three pan-CoV PCR assays in human samples; Table S3. Comparative results of three pan-CoV PCR assays in wastewater samples; Table S4. Comparative results of three pan-CoV PCR assays in bat samples; Table S5. Comparative results of three pan-CoV PCR assays in avian samples; Table S6. Number of primer mismatches found in each primer used in this study; Table S7. Test for Equality of Several Population Proportions (Q:W:X).
