# Supplementary material for: Comparative Performance in the Detection of Four Coronavirus Genera from Human, Animal, and Environmental Specimens
Source: Viruses. 2024 Mar 29;16(4):534. doi: 10.3390/v16040534 (PMC11054315; doi:10.3390/v16040534)
Supplement: Supplementary file 1 [file viruses-16-00534-s001.zip › viruses-2903173-supplementary.pdf]

Table S1. Pan-CoV primers and PCR protocol used in this study.

PCR name	PCR format	Primer's name	Sequence (5' - 3')	Protocol	Amplicon size (bp)	Targeted region *	Reference
**Q-CoV PCR**	Two-step RT-PCR	CoV-FWD1	CGTTGGIACWAAYBTVCCWYTICARBTRGG	95°C 5 min, then 15 cycles of 95°C for 30 seconds, 65°C for 30 seconds and 72°C for 45 seconds, then 35 cycles of 94°C for 30 seconds, 50°C for 30 seconds and 72°C for 45 seconds. Finish with 72°C for 7 minutes.	520	17,480-17,820.	Quan, PL. et al. [26]
		CoV-RVS1	GGTCATKATAGCRTCAVMASWWGCNACATG				
	Nested PCR	CoV-FWD2	GGCWCCWCCHGGNGARCAATT	Same protocol for rounds 1 and 2.	328		
		CoV-RVS2	GGWAWCCCCAYTGYTGWAYRTC				
**W-CoV PCR**	Two-step PCR	CoV-FWD3	GGTTGGGAYTAYCCHAARTGTGA	94°C 2 min, then 35 cycles of 94°C for 20 seconds, 50°C for 30 seconds and 72°C for 30 seconds. Finish with 72°C for 7 mins.	440	14,370- 14,750	Modified from Watanabe, S. et al. [29]
		CoV-RVS3	CCATCATCASWYRAATCATCATA				
	Hemi-nested	CoV-FWD4	GAYTAYCCHAARTGTGAYAGAGC	Same protocol for rounds 1 and 2.	434		
**X-CoV PCR**	Two-step PCR	pan-CoV_outF	CCAARTTYTAYGGHGGITGG	30 cycles of 94 °C for 15 s, 53.4 °C for 30 s and 68 °C for 1 min; a final extension at 68 °C for 5 min; and holding at 4 °C.	670-673	14255-14927	Xiu, L. et al. [21]
		pan-CoV_R	TGTTGIGARCARAAYTCATGIGG				
	Hemi-nested	pan-CoV_inF	GGTTGGGAYTAYCCHAARTGTGA	94 °C for 5 min; 35 cycles of 94 °C for 15 s, 54.5 °C for 30 s and 72 °C for 1 min; and a final extension of 72 °C for 10 min.	599-602		

* The position of PCR product compare with whole genome of HCoV-229E (Gen Bank accession No. NC_002645.1)

Table S2. Comparative results of three pan-CoV PCR assays in human samples.

Sample ID	Collection date	Sample type	Real-time PCR results		PCR and sequencing results tested by three pan-CoV PCR assays		
			Detected virus	Ct values	Q-CoV PCR	W-CoV PCR	X-CoV PCR
H001	20-Oct-22	Nasopharyngeal swab	HCoV-OC43	16.92	HCoV-OC43	HCoV-OC43	HCoV-OC43
H002	16-May-23	Nasopharyngeal swab	HCoV-OC43	19.9	HCoV-OC43	HCoV-OC43	HCoV-OC43
H003	4-Mar-23	Nasopharyngeal swab	HCoV-OC43	25.72	HCoV-OC43	HCoV-OC43	HCoV-OC43
H004	3-Jan-23	Nasopharyngeal swab	HCoV-OC43	27.44	HCoV-OC43	HCoV-OC43	HCoV-OC43
H005	1-Mar-23	Nasopharyngeal swab	HCoV-OC43	28.13	HCoV-OC43	HCoV-OC43	HCoV-OC43
H006	28-Jan-23	Nasal swab	HCoV-OC43	28.65	HCoV-OC43	Negative	Negative
H007	3-May-23	Nasopharyngeal swab	HCoV-OC43	31.16	HCoV-OC43	Negative	Negative
H008	28-Jan-23	Nasal swab	H-CoV 229E	12.8	H-CoV 229E	H-CoV 229E	H-CoV 229E
H009	28-Jan-23	Nasopharyngeal swab	H-CoV 229E	16.41	H-CoV 229E	H-CoV 229E	H-CoV 229E
H010	28-Jan-23	Nasal swab	H-CoV 229E	22.78	H-CoV 229E	H-CoV 229E	H-CoV 229E
H011	28-Jan-23	Nasopharyngeal swab	H-CoV 229E	27.15	H-CoV 229E	H-CoV 229E	H-CoV 229E
H012	3-May-23	Nasopharyngeal swab	H-CoV 229E	28.07	H-CoV 229E	Negative	H-CoV 229E
H013	15-May-23	Nasopharyngeal swab	HCoV HKU1	23.4	HCoV HKU1	Negative	HCoV HKU1
H014	26-Dec-23	Nasopharyngeal swab	SARS-CoV-2	24.71	SARS-CoV-2	Negative	SARS-CoV-2
H015	13-Jan-23	Nasopharyngeal swab	SARS-CoV-2	26.25	SARS-CoV-2	Negative	SARS-CoV-2
H016	25-Dec-23	Nasopharyngeal swab	SARS-CoV-2	26.88	SARS-CoV-2	Negative	SARS-CoV-2
H017	18-Aug-23	Nasopharyngeal swab	SARS-CoV-2	27.45	SARS-CoV-2	Negative	SARS-CoV-2
H018	10-Jan-23	Nasopharyngeal swab	SARS-CoV-2	29.86	SARS-CoV-2	Negative	SARS-CoV-2
H019	16-Jan-23	Nasopharyngeal swab	SARS-CoV-2	30.86	SARS-CoV-2	Negative	SARS-CoV-2
H020	20-Oct-22	Nasopharyngeal swab	SARS-CoV-2	29.4	SARS-CoV-2	Negative	SARS-CoV-2
H021	27-Sep-23	Nasopharyngeal swab	HCoV NL63	20.99	HCoV NL63	Negative	HCoV NL63
H022	18-Jul-23	Nasopharyngeal swab	HCoV NL63	29.51	Negative	Negative	HCoV NL63
H023	30-May-23	Nasopharyngeal swab	HCoV NL63	29.5	Negative	Negative	HCoV NL63
H024	19-Dec-22	Nasopharyngeal swab	Negative	Negative	Negative	Negative	Negative
H025	22-Dec-22	Nasopharyngeal swab	Negative	Negative	Negative	Negative	Negative
H026	20-Dec-22	Nasopharyngeal swab	Negative	Negative	Negative	Negative	Negative
H027	26-Dec-22	Nasopharyngeal swab + Throat swab	Negative	Negative	Negative	Negative	Negative
H028	26-Dec-22	Nasopharyngeal swab + Throat swab	Negative	Negative	Negative	Negative	Negative
H029	26-Dec-22	Nasopharyngeal swab + Throat swab	Negative	Negative	Negative	Negative	Negative
H030	27-Dec-22	Nasopharyngeal swab + Throat swab	Negative	Negative	Negative	Negative	Negative
H031	30-Dec-22	Nasopharyngeal swab + Throat swab	Negative	Negative	Negative	Negative	Negative
H032	30-Dec-22	Nasopharyngeal swab + Throat swab	Negative	Negative	Negative	Negative	Negative
H033	2-Jan-22	Nasopharyngeal swab + Throat swab	Negative	Negative	Negative	Negative	Negative
H034	4-Jan-22	Nasopharyngeal swab + Throat swab	Negative	Negative	Negative	Negative	Negative
H035	4-Jan-23	Nasopharyngeal swab + Throat swab	Negative	Negative	Negative	Negative	Negative
H036	8-Jan-23	Nasopharyngeal swab + Throat swab	Negative	Negative	Negative	Negative	Negative
H037	9-Jan-23	Nasopharyngeal swab + Throat swab	Negative	Negative	Negative	Negative	Negative
H038	9-Jan-23	Nasopharyngeal swab + Throat swab	Negative	Negative	Negative	Negative	Negative
H039	9-Jan-23	Nasopharyngeal swab + Throat swab	Negative	Negative	Negative	Negative	Negative
H040	9-Jan-23	Tracheal suction	Negative	Negative	Negative	Negative	Negative
H041	11-Jan-23	Nasopharyngeal swab + Throat swab	Negative	Negative	Negative	Negative	Negative
H042	20-Jan-23	Nasopharyngeal swab + Throat swab	Negative	Negative	Negative	Negative	Negative
H043	20-Jan-23	Nasopharyngeal swab + Throat swab	Negative	Negative	Negative	Negative	Negative
H044	20-Jan-23	Nasopharyngeal swab + Throat swab	Negative	Negative	Negative	Negative	Negative
H045	17-Jan-23	Nasopharyngeal swab + Throat swab	Negative	Negative	Negative	Negative	Negative
H046	17-Jan-23	Nasopharyngeal swab + Throat swab	Negative	Negative	Negative	Negative	Negative
H047	20-Jan-23	Nasopharyngeal and throat swab	Negative	Negative	Negative	Negative	Negative
H048	20-Jan-23	Nasopharyngeal and throat swab	Negative	Negative	Negative	Negative	Negative
H049	20-Jan-23	Tracheal suction	Negative	Negative	Negative	Negative	Negative
H050	8-Feb-23	Nasopharyngeal swab	Negative	Negative	Negative	Negative	Negative

Table S3. Comparative results of three pan-CoV PCR assays in wastewater samples.

Sample ID	Source	Collection date	Sample type	Real-time PCR	PCR ct values	PCR and sequencing results tested by three pan-CoV PCR assays		
						Q-CoV PCR	W-CoV PCR	X-CoV PCR
W01	Hospital	7-Jul-22	Watewater	Positive	28.08	SARS-CoV-2	Negative	SARS-CoV-2*
W02	Hospital	27-Sep-22	Watewater	Positive	29.39	SARS-CoV-2	Negative	SARS-CoV-2*
W03	Hospital	10-Apr-22	Watewater	Positive	25.59	SARS-CoV-2	Negative	SARS-CoV-2
W04	Hospital	22-Nov-22	Watewater	Positive	29.03	SARS-CoV-2	HCoV-OC43	SARS-CoV-2*
W05	Hospital	22-Nov-22	Watewater	Positive	29.57	SARS-CoV-2	HCoV-OC43	SARS-CoV-2*
W06	Hospital	13-Dec-22	Watewater	Positive	27.64	SARS-CoV-2	HCoV-229E	HCoV-229E
W07	Hospital	13-Dec-22	Watewater	Positive	29.68	SARS-CoV-2	HCoV-229E	HCoV-229E*
W08	Hospital	4-Apr-23	Watewater	Positive	25.48	SARS-CoV-2	Negative	SARS-CoV-2
W09	Hospital	18-Apr-23	Watewater	Positive	29.99	SARS-CoV-2	Negative	SARS-CoV-2
W10	Hospital	13-Dec-22	Watewater	Positive	32.22	HCoV-OC43	HCoV-OC43	HCoV-OC43*
W11	Hospital	25-Oct-22	Watewater	Positive	32.66	HCoV-OC43	HCoV-OC43	HCoV-OC43*
W12	Hospital	20-Dec-22	Watewater	Positive	31.54	HCoV-OC43	Negative	HCoV-OC43*
W13	Hospital	1-Mar-23	Watewater	Positive	32.47	HCoV-OC43	Negative	HCoV-OC43*
W14	Hospital	17-Jan-23	Watewater	Positive	30.49	HCoV-OC43	Negative	HCoV-OC43*
W15	Hospital	24-Jan-23	Watewater	Positive	33.23	HCoV-229E	Negative	HCoV-229E*
W16	Hospital	14-Mar-23	Watewater	Positive	34.40	HCoV-OC43	Negative	HCoV-OC43
W17	Hospital	25-Apr-23	Watewater	Positive	30.24	HCoV-OC43*	Negative	HCoV-OC43*
W18	Hospital	23-May-23	Watewater	Positive	36.62	HCoV-OC43	Negative	HCoV-OC43*
W19	Hospital	23-May-23	Watewater	Positive	33.37	HCoV-OC43	Negative	HCoV-OC43*
W20	Hospital	23-May-23	Watewater	Positive	31.25	HCoV-OC43	Negative	HCoV-OC43
W21	Hospital	23-May-23	Watewater	Positive	31.7	HCoV-OC43*	Negative	HCoV-OC43*
W22	BKK Airport-	18-Oct-23	Lavatory waste	Positive	30.3	Negative	Negative	SARS-CoV-2
W23	BKK Airport-	18-Oct-23	Lavatory waste	Positive	30.45	Negative	Negative	SARS-CoV-2*
W24	BKK Airport-	9-Nov-23	Lavatory waste	Positive	30.43	HCoV-OC43*	Negative	SARS-CoV-2*
W25	BKK Airport-	9-Nov-23	Lavatory waste	Positive	28.62	HCoV-OC43*	Negative	SARS-CoV-2
W26	BKK Airport-	19-Oct-23	Lavatory waste	Positive	33.1	Negative	Negative	Negative
W27	BKK Airport-	19-Oct-23	Lavatory waste	Positive	34.28	HCoV-229E	Negative	Negative
W28	Hospital	14/3/2023	Watewater	Negative	Negative	HCoV-229E	Negative	HCoV-OC43*
W29	Hospital	28/2/2023	Watewater	Negative	Negative	HCoV-OC43	Negative	HCoV-OC43*
W30	BKK Airport	15-Oct-23	Lavatory waste	Negative	Negative	HCoV-229E	Negative	Negative
W31	BKK Airport	27-Nov-23	Lavatory waste	Negative	Negative	HCoV-229E	Negative	Negative
W32	BKK Airport	27-Nov-23	Lavatory waste	Negative	Negative	HCoV-229E	Negative	Negative
W33	BKK Airport	27-Nov-23	Lavatory waste	Negative	Negative	HCoV-229E	Negative	Negative
W34	BKK Airport-	1-Oct-23	Lavatory waste	Negative	Negative	Negative	Negative	Negative
W35	BKK Airport-	1-Oct-23	Lavatory waste	Negative	Negative	Negative	Negative	Negative
W36	BKK Airport-	3-Oct-23	Lavatory waste	Negative	Negative	Negative	Negative	Negative
W37	BKK Airport-	6-Oct-23	Lavatory waste	Negative	Negative	Negative	Negative	Negative
W38	BKK Airport-	6-Oct-23	Lavatory waste	Negative	Negative	Negative	Negative	Negative
W39	BKK Airport-	6-Oct-23	Lavatory waste	Negative	Negative	Negative	Negative	Negative
W40	BKK Airport-	6-Oct-23	Lavatory waste	Negative	Negative	Negative	Negative	Negative
W41	BKK Airport-	15-Oct-23	Lavatory waste	Negative	Negative	Negative	Negative	Negative
W42	BKK Airport-	18-Oct-23	Lavatory waste	Negative	Negative	Negative	Negative	Negative
W43	BKK Airport-	18-Oct-23	Lavatory waste	Negative	Negative	Negative	Negative	Negative
W44	BKK Airport-	31-Oct-23	Lavatory waste	Negative	Negative	Negative	Negative	Negative
W45	BKK Airport-	31-Oct-23	Lavatory waste	Negative	Negative	Negative	Negative	Negative
W46	BKK Airport-	31-Oct-23	Lavatory waste	Negative	Negative	Negative	Negative	Negative

*The overlapped nucleotide peaks were found in the chromatogram, the major peaks were called for CoV species identification.

Table S4. Comparative results of three pan-CoV PCR assays in bat samples.

Sample ID	Animal species	Collection date	Sample type	PCR and sequencing results tested by three pan-CoV PCR assays		
				**Q-CoV PCR**	**W-CoV PCR**	**X-CoV PCR**
B02	*Hipposideros pomona*	22/6/2020	Rectal swab	Coronavirus HKU10	Coronavirus HKU10	Coronavirus HKU10
B01	*Hipposideros larvatus*	22/6/2020	Rectal swab	Alphacoronavirus HlYN10	Alphacoronavirus HlYN10	Alphacoronavirus HlYN10
B03	*Hipposideros larvatus*	22/6/2020	Rectal swab	Alphacoronavirus HlYN10	Alphacoronavirus HlYN10	Alphacoronavirus HlYN10
B04	*Hipposideros larvatus*	22/6/2020	Rectal swab	Alphacoronavirus HlYN10	Alphacoronavirus HlYN10	Alphacoronavirus HlYN10
B05	*Hipposideros larvatus*	22/6/2020	Rectal swab	Alphacoronavirus HlYN10	Alphacoronavirus HlYN10	Alphacoronavirus HlYN10
B06	*Hipposideros larvatus*	23/6/2020	Rectal swab	Alphacoronavirus HlYN10	Alphacoronavirus HlYN10	Alphacoronavirus HlYN10
B07	*Hipposideros larvatus*	23/6/2020	Rectal swab	Alphacoronavirus HlYN10	Alphacoronavirus HlYN10	Alphacoronavirus HlYN10
B08	*Hipposideros larvatus*	23/6/2020	Rectal swab	Alphacoronavirus HlYN10	Alphacoronavirus HlYN10	Alphacoronavirus HlYN10
B09	*Hipposideros larvatus*	23/6/2020	Rectal swab	Alphacoronavirus HlYN10	Alphacoronavirus HlYN10	Alphacoronavirus HlYN10
B10	*Hipposideros larvatus*	23/6/2020	Rectal swab	Alphacoronavirus HlYN10	Alphacoronavirus HlYN10	Alphacoronavirus HlYN10
B11	*Hipposideros larvatus*	23/6/2020	Rectal swab	Alphacoronavirus HlYN10	Alphacoronavirus HlYN10	Alphacoronavirus HlYN10
B12	*Hipposideros larvatus*	23/6/2020	Rectal swab	Alphacoronavirus HlYN10	Alphacoronavirus HlYN10	Alphacoronavirus HlYN10
B14	*Rhinolophus spp.*	22/6/2020	Rectal swab	Alphacoronavirus HlYN10	Not detected	Alphacoronavirus YN2012
B16	*Rhinolophus spp.*	23/6/2020	Rectal swab	Alphacoronavirus HlYN10	Not detected	Not detected
B13	*Rhinolophus spp.*	22/6/2020	Rectal swab	Alphacoronavirus RsYN14	Alphacoronavirus RsYN14	Alphacoronavirus RsYN14
B15	*Rhinolophus spp.*	22/6/2020	Rectal swab	Alphacoronavirus RsYN14	Alphacoronavirus RsYN14	Alphacoronavirus RsYN14
B17	*Rhinolophus spp*	22/6/2020	Rectal swab	Sarbecovirus Ra22QT77	Not detected	Sarbecovirus Ra22QT77
B18	*Rhinolophus spp*	22/6/2020	Rectal swab	Sarbecovirus Ra22QT77	Not detected	Not detected
B19	*Rhinolophus spp*	22/6/2020	Rectal swab	Sarbecovirus Ra22QT77	Not detected	Sarbecovirus Ra22QT77
B20	*Rhinolophus spp*	22/6/2020	Rectal swab	Sarbecovirus Ra22QT77	Not detected	Alphacoronavirus HlYN10
B21	*Rhinolophus spp*	22/6/2020	Rectal swab	Sarbecovirus Ra22QT77	Not detected	Sarbecovirus Ra22QT77
B22	*Rhinolophus spp*	22/6/2020	Rectal swab	Sarbecovirus Ra22QT77	Alphacoronavirus RaYN17	Sarbecovirus Ra22QT77
B23	*Rhinolophus spp*	22/6/2020	Rectal swab	Sarbecovirus Ra22QT77	Alphacoronavirus RaYN17	Sarbecovirus Ra22QT77
B24	*Rhinolophus spp*	23/6/2020	Rectal swab	Sarbecovirus Ra22QT77	Not detected	Sarbecovirus Ra22QT77
B25	*Rhinolophus spp*	23/6/2020	Rectal swab	Sarbecovirus Ra22QT77	Not detected	Sarbecovirus Ra22QT77
B26	*Rhinolophus spp*	23/6/2020	Rectal swab	Sarbecovirus Ra22QT77	Not detected	Sarbecovirus Ra22QT77
B27	*Rhinolophus spp*	23/6/2020	Rectal swab	Sarbecovirus Ra22QT77	Not detected	Sarbecovirus Ra22QT77
B28	*Rhinolophus spp*	23/6/2020	Rectal swab	Sarbecovirus Ra22QT77	Not detected	Sarbecovirus Ra22QT77

Table S5. Comparative results of three pan-CoV PCR assays in avian samples.

Sample ID	Host	Collection date	Sample type	PCR and sequencing results tested by three pan-CoV PCR assays		
				X-CoV PCR	Q-CoV PCR	W-CoV PCR
A01	Whiskered Tern	28-Oct-20	Rectal swab	Avian coronavirus Glaucous-winged gull CIR-66002	Negative	Negative
A02	Pigeon	12-Mar-21	Oral swab	Pigeon-dominant Coronavirus	Pigeon-dominant Coronavirus	Negative
A03	Lesser Whistling-duck	18-Mar-21	Rectal swab	Anser fabalis coronavirus NCN2	Negative	Anser fabalis coronavirus NCN2
A04	Lesser Whistling-duck	18-Mar-21	Rectal swab	Anser fabalis coronavirus NCN2	Negative	Anser fabalis coronavirus NCN2
A05	Lesser Whistling-duck	18-Mar-21	Oral swab	Anser fabalis coronavirus NCN2	Negative	Anser fabalis coronavirus NCN2
A06	Little Egret	29-Sep-21	Rectal swab	Avian coronavirus isolate CoV-9518-2016	Negative	Avian coronavirus isolate CoV-9518-2016
A07	Little Egret	29-Sep-21	Oral swab	Avian coronavirus isolate CoV-9518-2016	Negative	Negative
A08	Cattle Egret	12-Mar-21	Rectal swab	Unclassified Deltacoronavirus sp.	Negative	Negative
A09	Cattle Egret	12-Mar-21	Rectal swab	Night-heron coronavirus HKU19	Negative	Negative
A10	Cattle Egret	12-Mar-21	Rectal swab	Unclassified Deltacoronavirus sp.	Negative	Negative
A11	Cattle Egret	12-Mar-21	Rectal swab	Unclassified Deltacoronavirus sp.	Negative	Negative
A12	Cattle Egret	12-Mar-21	Rectal swab	Unclassified Deltacoronavirus sp.	Negative	Negative
A13	Cattle Egret	12-Mar-21	Rectal swab	Unclassified Deltacoronavirus sp.	Negative	Negative
A14	Indian Cormorant	12-Mar-21	Rectal swab	Unclassified Deltacoronavirus sp.	Negative	Negative
A15	Indian Cormorant	12-Mar-21	Rectal swab	Unclassified Deltacoronavirus sp.	Negative	Negative
A16	House Swallow	21-Dec-20	Rectal swab	Magpie-robin coronavirus HKU18	Negative	Negative
A17	House Swallow	21-Dec-20	Oral swab	Magpie-robin coronavirus HKU18	Negative	Negative

Table S6. Number of primer mismatches found in each primer used in this study.

Coronavirus	No. of reference WGS used*	CoV PCR protocol -Primer name<br>No. of Primer mismatches (number of mapped CoV WGS)									
		Q-FWD1	Q-FWD2	Q-RVS1	Q-RVS2	W-FWD3	FWD4	RVS3	X-OutF	X-InF	X-R
HCoV-HKU1	85	3 (81)	2 (36)	4 (47)	1 (47)	0 (84)	1 (84)	0 (82)	0 (84)	0 (84)	0 (83)
		14 (3)	4 (47)	5 (38)	2 (38)	8 (1)	10 (1)	7 (3)	1 (1)	1 (1)	9 (1)
		15 (1)	7 (1)								11 (1)
			8 (1)								
HCoV-NL63	158	3 (1)	2 (154)	4 (12)	2 (157)	0 (158)	1 (157)	7 (158)	0 (158)	0 (158)	1 (158)
		4 (151)	3 (3)	5 (145)	4 (1)		2 (1)				
		5 (5)	9 (1)	6 (1)							
		11 (11)									
HCoV-229E	117	2 (117)	1 (117)	2 (7)	2 (117)	1 (24)	1 (24)	1 (110)	0 (116)	1 (24)	1 (116)
				3 (109)		2 (92)	2 (92)	2 (6)	5 (1)	2 (92)	2 (1)
				4 (1)		6 (1)	6 (1)	10 (1)		6 (1)	
HCoV-OC43	331	1 (308)	1 (329)	2 (337)	2 (330)	0 (328)	1 (330)	0 (330)	1 (330)	0 (328)	0 (317)
		2 (21)	2 (1)	3 (2)	7 (1)	1 (3)	2 (1)	12 (1)	3 (1)	1 (3)	1 (14)
		13 (2)	4 (1)	11 (2)							
MERS-CoV	342	16 (342)	3 (342)	4 (342)	2 (342)	0 (341)	1 (341)	2 (331)	1 (341)	0 (341)	0 (1)
						10 (1)	9 (1)	3 (9)	8 (1)	10 (1)	1 (340)
								10 (2)			7 (1)
SARS-CoV	8	2 (8)	4 (8)	1 (8)	3 (8)	0 (8)	1 (8)	9 (8)	1 (8)	0 (8)	2 (8)
SARS-CoV-2	200	1 (1)	4 (198)	2 (197)	2 (199)	0 (200)	1 (200)	6 (1)	1 (199)	0 (200)	0 (200)
		2 (196)	5 (1)	3 (3)	3 (1)			7 (1)	2 (1)		
		5 (3)	6 (1)					8 (139)			
								11 (56)			
								12 (3)			
Bat Alphacoronavirus	134	0 (2)	1 (38)	0 (4)	0 (2)	0 (84)	1 (120)	5 (13)	0 (24)	0 (84)	0 (83)
		1 (11)	2 (78)	1 (19)	1 (59)	1 (43)	2 (11)	6 (13)	1 (110)	1 (43)	1 (49)
		2 (64)	3 (17)	2 (36)	2 (51)	2 (7)	3 (3)	7 (26)		2 (7)	2 (2)
		3 (36)	8 (1)	3 (18)	3 (20)			8 (5)			
		4 (13)		4 (9)	4 (2)			9 (7)			
		5 (6)		5 (21)				10 (19)			
		16 (1)		6 (24)				11 (39)			
		17 (1)		9 (3)				12 (12)			
Bat Betacoronavirus	263	1 (22)	0 (1)	1 (125)	1 (11)	0 (245)	1 (229)	4 (2)	0 (3)	0 (245)	0 (35)
		2 (167)	1 (6)	2 (71)	2 (159)	1 (17)	2 (33)	5 (5)	1 (231)	1 (17)	1 (111)
		3 (40)	2 (26)	3 (19)	3 (77)	2 (1)	3 (1)	6 (7)	2 (29)	2 (1)	2 (117)
		4 (1)	3 (114)	4 (28)	4 (16)			7 (15)			
		5 (1)	4 (113)	5 (7)				8 (23)			
		9 (2)	5 (3)	6 (5)				9 (86)			
		10 (2)		7 (8)				10 (99)			
		15 (3)						11 (14)			
		16 (13)						12 (8)			
		17 (7)						13 (4)			
		18 (4)									
		19 (1)									
Gamma-CoV	696	1 (194)	0 (21)	1 (12)	1 (494)	0 (632)	1 (560)	5 (23)	0 (345)	0 (632)	0 (170)
		2 (134)	1 (538)	2 (88)	2 (170)	1 (35)	2 (106)	6 (64)	1 (322)	1 (35)	1 (473)
		3 (326)	2 (68)	3 (240)	3 (1)	7 (1)	3 (1)	7 (13)	5 (1)	7 (1)	2 (21)
		4 (37)	3 (62)	4 (337)	4 (2)	8 (15)	7 (1)	11 (161)	6 (11)	8 (15)	3 (1)
		5 (8)	4 (10)	5 (19)	6 (3)	9 (1)	8 (20)	13 (347)	7 (2)	9 (1)	6 (1)
Delta-CoV	36	1 (6)	2 (6)	2 (12)	6 (3)	0 (26)	2 (24)	5 (8)	0 (35)	0 (26)	0 (16)
		2 (6)	3 (18)	3 (8)	7 (11)	1 (10)	3 (12)	8 (4)	1 (1)	1 (10)	1 (20)
		3 (7)	4 (8)	4 (4)	8 (5)			10 (6)			
		4 (7)	5 (2)	5 (8)	9 (13)			11 (11)			
		5 (8)	6 (2)	6 (2)	10 (2)			13 (7)			

*Whole genome sequences of coronavirus were download from NCBI Gen Bank (accessed on 7 January 2024) and used for primer mapping.
